# Establishing comprehensive quaternary structural proteomes from genome sequence

**DOI:** 10.21203/rs.3.rs-2923626/v1

**Published:** 2023-05-19

**Authors:** Edward Alexander Catoiu, Nathan Mih, Maxwell Lu, Bernhard Palsson

**Affiliations:** 1 Department of Bioengineering, University of California, San Diego, La Jolla, CA 92101; 2 Omnicorp Inc. (Pilot AI), San Francisco, CA 94129; 3 The Novo Nordisk Foundation (NNF) Center for Biosustainability, The Technical University of Denmark, Kongens Lyngby 2800, Denmark

## Abstract

A critical body of knowledge has developed through advances in protein microscopy, protein-fold modeling, structural biology software, availability of sequenced bacterial genomes, large-scale mutation databases, and genome-scale models. Based on these recent advances, we develop a computational platform that; i) computes the oligomeric structural proteome encoded by an organism’s genome; ii) maps multi-strain alleleomic variation, resulting in the structural proteome for a species; and iii) calculates the 3D orientation of proteins across subcellular compartments with angstrom-level precision. Using the platform, we; iv) compute the full quaternary *E. coli* K-12 MG1655 structural proteome; v) deploy structure-guided analyses to identify consequential mutations; and, in combination with a genome-scale model that computes proteome allocation, vi) obtain a draft 3D visualization of the proteome in a functioning cell. Thus, in conjunction with relevant datasets and computational models, we can now resolve genome-scale structural proteomes to obtain an angstrom-level understanding of whole-cell functions.

## Introduction

The proteome of the cell is responsible for metabolite uptake and secretion, genetic information processing and replication, energy production, and all other processes required for maintaining cellular homeostasis. Before becoming a functional unit of this multi-scale system, a protein must fold properly into its native three-dimensional shape. This folding process is also multiscale. A peptide sequence (primary sequence) associates locally to form small recognizable patterns (e.g., alpha-helices and beta-sheets). Often stabilized by disulfide bridges and physiochemical attraction, these secondary structures fold onto each other to form larger recognizable domains, resulting in the three-dimensional structure of the protein monomer (tertiary structure). These protein monomers often oligomerize and form multi-subunit enzymes (quaternary structures) that carry out the functions in the cell.

Structural biology—the study of protein shape and function—has advanced rapidly in recent years. For proteins involved in large quaternary enzyme complexes and for those spanning the cell membrane, the three-dimensional shape was particularly difficult to study with classical crystallographic techniques. The development of cryogenic electron microscopy – a method that images thin slices of a protein frozen in its native state (much like a biopsy)—has drastically increased the speed and ease by which these previously unknowable protein structures can be resolved^1–5^. Concurrently, computational methods have also experienced increasing success in accurately predicting protein structures^6–10^. Most recently, deep learning algorithms^11–12^ (e.g., AlphaFold) have utilized multiple sequence alignments and incorporated biophysical knowledge about protein structure to confidently predict the shape of proteins without homologous structures in the Protein Data Bank^13^. Even more promising, these algorithms can be “hacked” to predict the structures of oligomeric assemblies of protein complexes^14^.

Although structural biology can offer molecular insights into a protein’s shape and function, mutations in key domains can change the enzymatic properties and modulate a protein’s function. Changes in protein function can be either beneficial (e.g., an increase in stability of the active form) or detrimental (e.g., a loss of substrate binding efficiency). Protein engineering employs a variety of techniques to find mutations that produce a desired phenotype. One such technique, multiplex automated genomic engineering (MAGE)^15^, can introduce many mutations with unknown effects at specific sites in the genome. Mutations that result in the desired phenotype can then be selected for. Laboratory evolution, an experimental approach involving the serial passage of a cell population in an increasingly stringent selection pressure, can speed up the evolutionary process and beneficial mutations can be identified by sequencing the endpoint strain^16–18^. The dramatic decrease in sequencing costs in the last ten years has allowed for many mutations identified by these experimental techniques to be collected in databases.

Concurrent with advancements in structural biology and the formation of large-scale mutation databases, systems biology—the study of systems-level cellular behavior— was driven by the development of genome-scale models (GEMs) of cellular metabolism that predict gene essentiality, growth phenotypes, and proteome allocation in a few organisms^19–24^. Software (e.g., ssbio^25^) was developed to map structural information to these modeled proteomes. Structural systems biology—the study of structural biology at the systems-level—has incorporated protein structures into genome-scale models (GEM-PROs) to study protein-fold evolution and investigate structural differences between organisms^26–30^. Notwithstanding the incorporation of protein information at the monomer-level, the use of GEM-PROs is the most recent step towards building genome-scale models that represent the physical nature of the cellular proteome.

Given the availability of high-quality protein structures and structural models that capture the shape of complex multi-subunit enzymes, the deposition of mutation-phenotype information into large-scale databases, and the development of genome-scale models of cellular proteome allocation, the creation of a genome annotation platform with interoperability between structural, functional, mutational, and systems-level information is now possible.

In this study, we present the Quaternary Structural Proteome Atlas of a CEll (QSPACE) at Genome-Scale (-GS). QSPACE-GS is a computational platform that 1) utilizes state-of-the-art modeling software (e.g. Alphafold^11^ & Alphafold Multimer^14^) and the latest crystallographic depositions to create a three-dimensional structural representation that accounts for the multi-subunit assembly of the cellular proteome; 2) calculates structural properties of the proteome; 3) provides a three-dimensional context to map functional information including enzymatic domains, binding sites, and protein interfaces; 4) draws mutational information from large-scale databases of laboratory-acquired mutations^18,31–33^ and of the wild-type natural sequence diversity (alleleome) of *E. coli*^34^; and 5) calculates the subcellular compartmentalization of the proteome with angstrom-level resolution.

We demonstrate how the interoperability of multiple data types provided by QSPACE can be used for biological discovery. Finally, we incorporate the protein structures selected by the QSPACE to generate a GEM-PRO that predicts the spatial allocation of the physical proteome across multiple subcellular compartments of *Escherichia coli* K-12 MG1655 at optimal growth rate. To our knowledge, this QSPACE/GEM-PRO is the most comprehensive whole-cell approach that captures the 3D nature of the *E. coli* structural proteome. As structural, mutational, and functional knowledge is discovered, and GEMs are developed with increasing specificity, QSPACE can provide a method to compile all information related to the structural proteome for an increasing number of organisms. QSPACE is available at https://github.com/EdwardCatoiu/QSPACE/

## Results

### 3D oligomeric structural representation of the proteome

**Figure 1:**
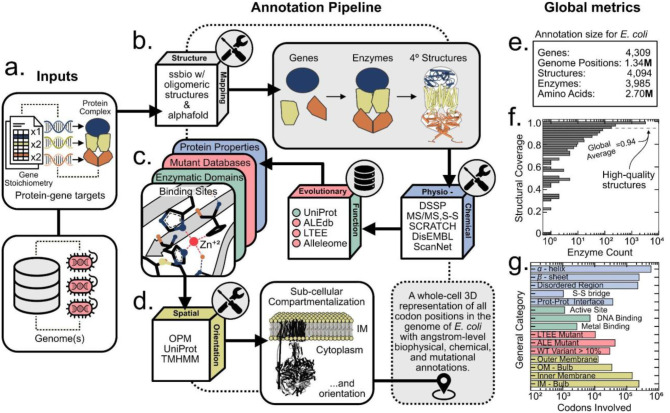
The Quaternary Structural Proteome Atlas of a CEll calculates the whole-cell 3D oligomeric structural representation of the *E. coli* proteome. **(a)** The pipeline takes two inputs: a genome (or list of genomes, optional) and a list of protein complexes with genes defined in a known stoichiometric ratio. This mapping will serve as the protein-gene target to be recreated by the **(b)** structure mapping module. Homology models from SWISS-MODEL, ITASSER, and Alphafold are combined with oligomeric (if applicable) PDB depositions, and *de novo* generated Alphafold Multimer models to find the best structure (or combinations of structures) that can recreate gene stoichiometry of the protein-target (more details in Figure 2). At the oligomeric level (if applicable), the protein structures **(c)** are analyzed using various software packages to calculate their physicochemical properties and to identify evolutionary variable regions and functional domains. **(d)** The structures are localized to their subcellular compartments and membrane-embedded structures are oriented with angstrom-level precision resulting in a three-dimensional representation of **(e)** the functional proteome of *E. coli*. The oligomeric nature of the proteome yields a 2:1 ratio between unique amino acid positions (on a specific protein subunit) and unique genomic positions. **(f)** The high-quality structural coverage of the *E. coli* proteome can be used to display **(g)** amino acid residues of importance to various physio-chemical, metabolic (functional), evolutionary, or spatial processes in three dimensions.

**Figure 2:**
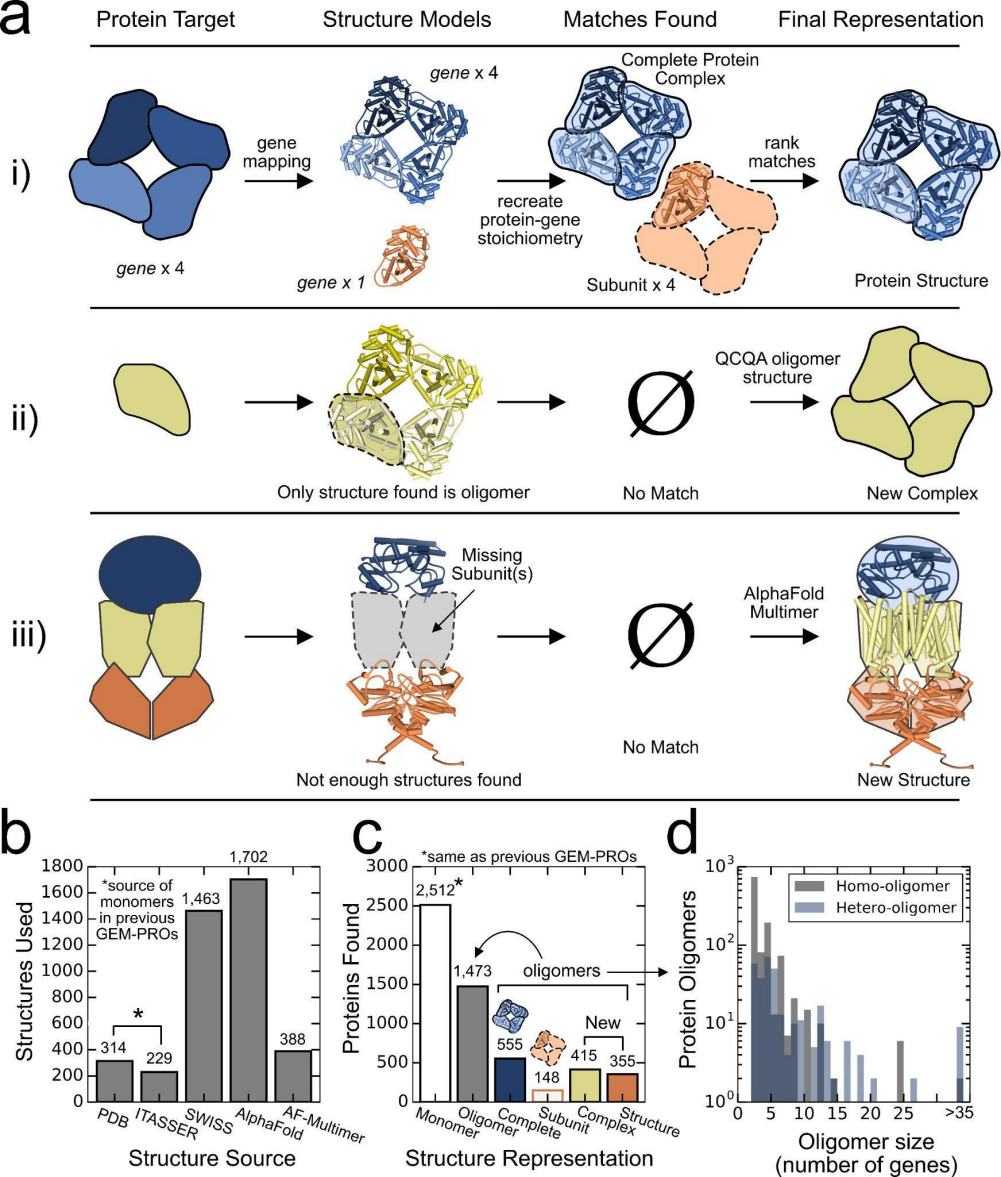
The protein-to-structure module yields a 3D structural representation that reflects the oligomeric nature of multi-subunit proteins. (**a**) There are three ways that the protein-structure module finds the 3D structural representation for a multi-subunit protein. (i) For each provided protein-gene stoichiometric target (left), all structures that share genes with the protein are identified (center left) and combined (if applicable) to recreate the protein-gene target (center right). If multiple matches are found, the structure that most accurately reflects the complete oligomeric enzyme is selected (right). (ii) Sometimes, the only structures that share genes with the protein-target contain more copies of the gene than are present in the protein-target. After QCQA inspection (Fig. S9), passing structures are used to redefine a new gene stoichiometry for 425 protein complexes (Dataset S2B). (iii) If the structures identified cannot recreate the protein in its entirety (missing subunits), Alphafold Multimer is used to fold the entire amino acid sequence of the enzyme oligomer (<2000 AAs) and return a *de novo* 3D model for 431 protein complexes (Fig. S10, Dataset S9). (**b**) To achieve a multi-subunit representation of the *E. coli* proteome, structures or models from various sources are used. This includes monomeric and k-meric Alphafold structures which have not been utilized in previous GEM-PRO models. (**c**) Unlike previous GEM-PROs, the protein-to-structure module yields 3D structures that represent the oligomeric nature of the *E. coli* proteome. This representation is further improved by using structural data to correct existing protein-gene stoichiometry and by Alphafold Multimer to calculate novel quaternary protein structures, (**d**) allowing for a truer accounting of the oligomeric architecture of the *E. coli* proteome.

The Quaternary Structural Proteome Atlas of a CEll at Genome-Scale (QSPACE-GS) generates a 3D representation of all codon positions in a genome – complete with angstrom-level biophysical, chemical, and mutational data (Dataset S1, see TABLE S1 for details). QSPACE takes two inputs (Fig. 1a): i) a dictionary of protein complexes and stoichiometric ratio of the genes that make up each protein complex (Dataset S2) and ii) an optional list of genomes (required for mapping wild-type sequence variation). Using a modified version of ssbio^25^, databases of monomeric homology models (ITASSER^8^, SWISS^10^ & AlphaFold^12^), and an external Google Colab notebook running AlphaFold Multimer^14^ (ColabFold^35^), we determine the 3D coordinate file (hereafter referred to as a “structure”) that most accurately reflects the multi-subunit protein assembly (Fig. 1b, details in Fig. 2).

**Figure 3:**
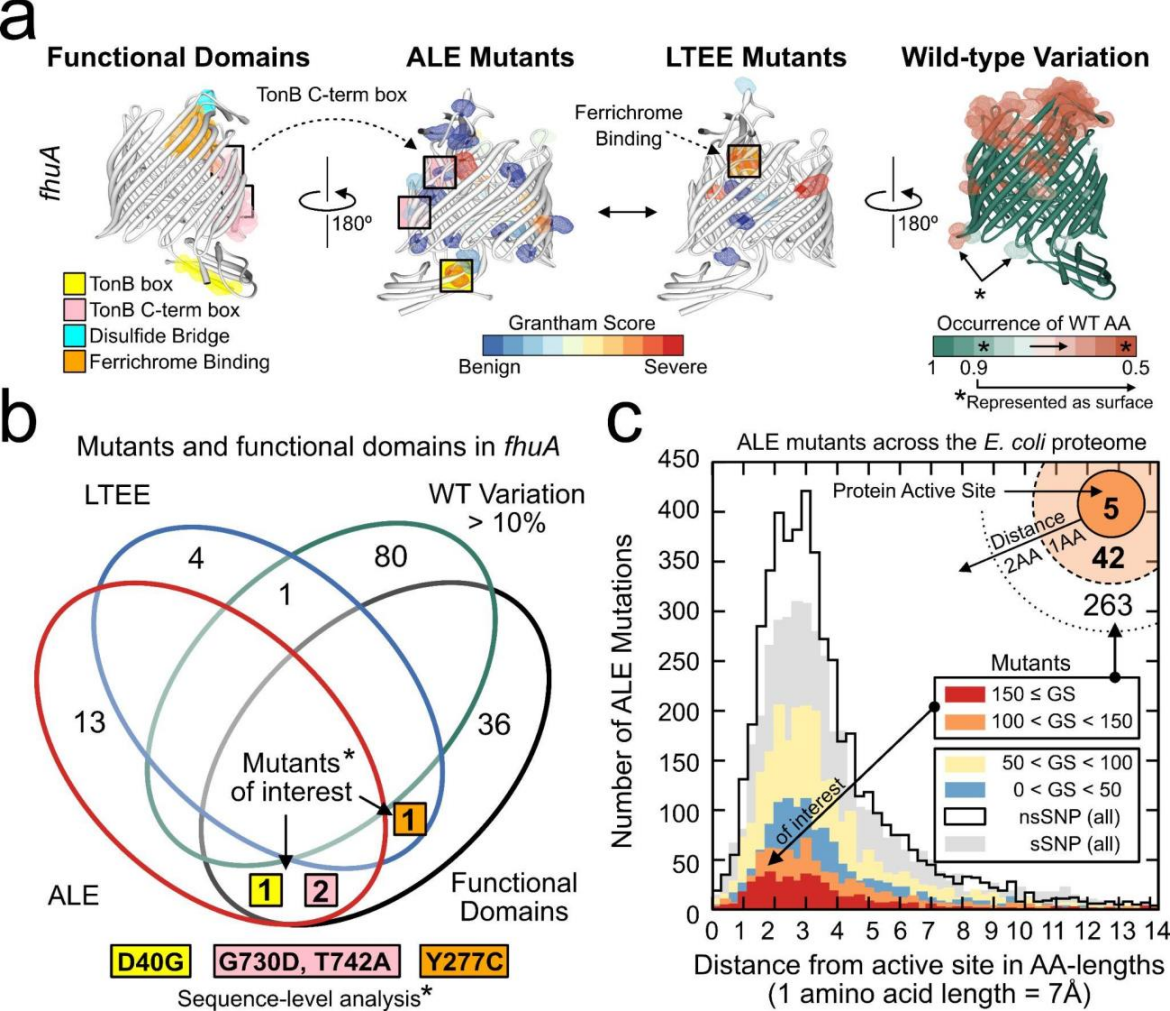
Multi-level mapping of functional and mutational datasets can help identify mutations likely to impact protein function. **(a)** Functional domains, mutants found in adaptive laboratory evolutions (ALEs) or in the long-term evolution experiment (LTEE), and wild-type variants (occurrence > 10%) are mapped onto the protein structure of the TonB-dependent outer membrane transporter *fhuA* (PDB: 2GRX). The overlay of these datasets (sequence-level) **(b)** can identify *fhuA* mutations that are likely to impact protein function or stability (severe mutations located within key regions), and mutations that are shared across natural and laboratory strains. **(c)** Proximity-based analysis of all ALE mutants and annotated active site residues reveals mutants of interest across the *E. coli* proteome. The inset (top right) counts the number of moderate-to-severe mutations located at various distances from annotated active sites. This analysis can be expanded for all key domains (protein-protein interfaces, binding sites, etc., see Fig. S4–6).

**Figure 4:**
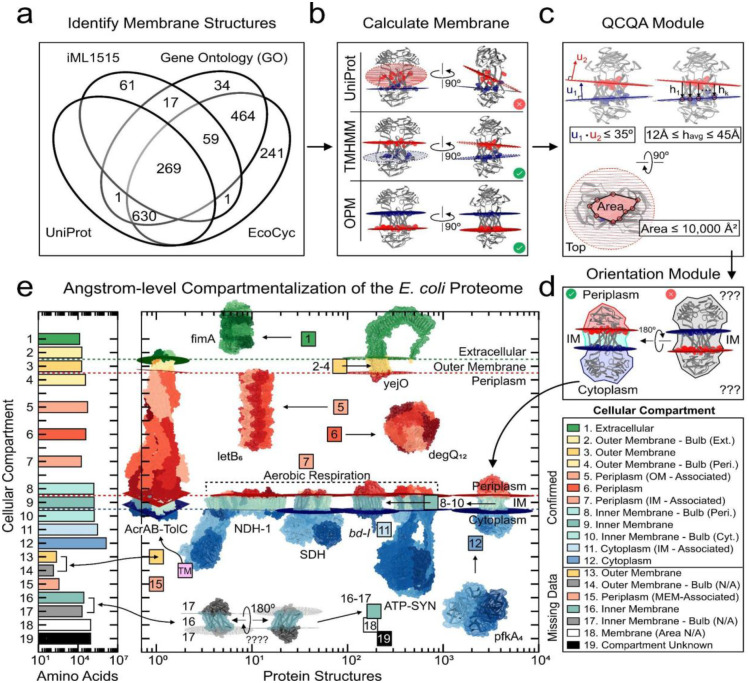
The membrane module yields angstrom-level subcellular compartmentalization of the *E. coli* proteome. **(a)** In the metadata provided by EcoCyc (cellular compartment), Gene Ontology Terms (pathway, function, compartment), iML1515^36^ (metabolic subsystem), and UniProt (topological & transmembrane domains) databases, there are 1,777 protein structures mapped to at least one gene that is associated with the *E. coli* membrane. **(b)** Membrane-crossing residues are identified by the amino acid sequence information provided by UniProt, predicted by DeepTMHMM, and calculated by OPM. From these residues, a plane of best fit is calculated. **(c)** Structures with two calculated membrane planes pass the QCQA analysis if i) the angle between the planes is less than 35°, ii) the thickness of the membrane embedded region is between 12 and 45 Angstroms, and iii) the cross-sectional area of the membrane embedded region is less than 10,000 Å^2^. **(d)** Membrane proteins are oriented using the topological information provided by UniProt (if available) or manually using common protein motifs (see Dataset S6-S7) such that **(e)** the subcellular compartment of every amino acid of the *E. coli* proteome can be determined.

**Figure 5:**
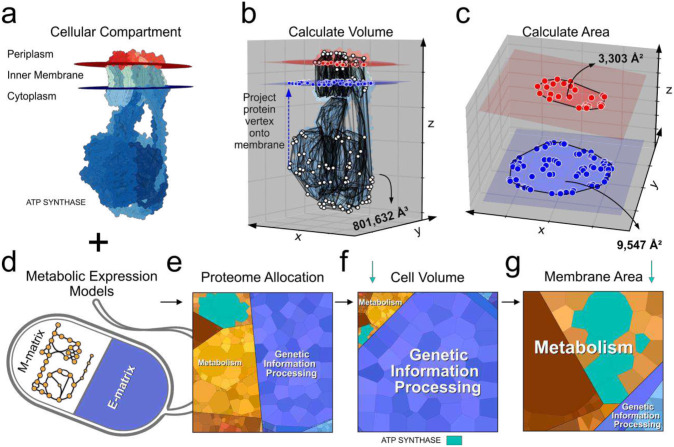
QSPACE integrates with genome-scale models to predict the physical space required by the *E. coli* proteome at optimal growth rate. **(a)** The compartmentalization of each amino acid of the *E. coli* proteome allows for the calculation of geometric properties of all proteins. **(b)** The volume of ATP-synthase and **(c)** its cross-sectional membrane area are shown. **(d)** The integration of QSPACE with genome-scale models (iJL1678b-ME^24^, in this case) of metabolism (M-matrix) and macromolecular expression (E-matrix) (ME-models) can be used to calculate **(e)** the proteome allocation, **(f)** the volumetric allocation, and **(g)** the membrane composition of *E. coli* at optimal growth rate. The expression, volume, and membrane area allocated to ATP-synthase is shown (Panels E-G, cyan). The calculated spatial allocation (Panels F-G) of the macromolecular expression predicted by existing ME-models (in Panel E) is a fundamental advancement towards building genome-scale biophysical whole-cell models.

Once the structure file representing the quaternary assembly of each protein is determined, multiple software packages and databases are used to map physio-chemical, evolutionary, and functional information onto the protein structures (Fig. 1c). The 3D overlay of multiple data types creates potential for many analysis tools (see Fig. 3). The amino acids in each protein are then assigned to one of twelve subcellular compartments; and those representing the membrane fraction of the proteome are oriented across one of the *E. coli* membranes (Fig. 1d, details in Fig. 4). These structures can be integrated with genome-scale systems models to yield a 3D understanding of the biophysical/spatial allocation of the proteome in a functioning cell (see Fig. 5).

As an example, we apply QSPACE to *E. coli* K-12 MG1655 and determine the quaternary structural representation of its proteome (Fig. 1e, Dataset S1). QSPACE achieves high quality coverage of the *E. coli* enzymes (Fig. 1f, Dataset S3) by selecting from both experimentally resolved structures that are deposited in the Protein DataBank (PDB)^13^ and from structural models calculated using state-of-the-art protein-fold modeling methods (ITASSER^8^, SWISS^10^, AlphaFold^12^ & AlphaFold Multimer^14^) (see Fig. 2b).

The set of structures that QSPACE uses to resolve the *E. coli* structural proteome can be used as 3D scaffolds to map multiple structural, functional, mutational, and spatial data types (Fig. 1g) with amino acid-level resolution.

### 3D structural representation of multi-subunit oligomeric enzymes

Multimeric proteins require oligomerization to function properly. The fundamental advancement (compared to iML1515-GP^36^ in Fig. S1, a comprehensive list is presented in Fig. S2) of the QSPACE over existing structural proteome formulations is that it captures the quaternary structural representation of multi-subunit proteins.

To ensure that the multi-subunit protein assembly is accurately reflected in the structural data, we designed the pipeline to identify the correct protein structure for a target oligomeric protein (Fig. 2a.i), to redefine a target protein’s gene stoichiometry when the structural data suggests oligomerization (Fig, 2a.ii), and to generate *de novo* structural models for oligomeric enzymes whose subunits cannot be fully represented by the structures in the PDB (Fig. 2a.iii).

QSPACE yields a structural representation of the *E. coli* functional proteome using structures deposited in the PDB and predicted models generated by SWISS-MODEL, I-TASSER, AlphaFold, and AlphaFold Multimer (Fig. 2b). The incorporation of AlphaFold and Alphafold Multimer structures (1,702 and 388 structures, respectively) offers higher quality coverage for 51.5% (2,053/3,985) of the *E. coli* protein complexes (Fig. S3). The collection of structures identified by QSPACE accurately captures the multi-subunit assembly of 1,473 oligomeric proteins (Fig. 2c). Proteins that are not known to oligomerize and that have no structural evidence of oligomerization are also mapped to their respective monomeric structures as in previous GEM-PRO formulations. We show that QSPACE captures the structural representation of oligomeric enzymes whose sizes can be quite large at the genome-scale (Fig. 2d). When compared to the latest *E. coli* genome-scale model with protein structures (iML1515-GP^36^), QSPACE advances the oligomeric structural annotation for 70% of genes in iML1515, while offering a 2.86-fold increase in gene coverage and higher quality structures (Fig. S1). To our knowledge, the result is the first full quaternary structural proteome for an organism and *de facto* represents a major advancement in genome annotation.

### Multi-level mapping of functional and mutational datasets helps identify mutations likely to impact protein function

An accurate 3D structural representation of the proteome can serve as a scaffold for mapping multiple data types, thus providing a structured approach to data integration. The interoperability of multiple datatypes can accelerate our understanding of structure-function relationships and mechanisms, as illustrated in Fig 3.

Here, we demonstrate QSPACE’s ability to identify mutations likely to affect the protein function of a single enzyme and to identify mutations of interest across the entire proteome. We first show all the domains of known enzymatic significance on the protein structure of the outer membrane transporter *fhuA* (Fig. 3a, left). Then, we can map the wild-type alleleomic variation^34^ onto the structure, as well as mutations acquired in adaptive laboratory evolution (ALE) experiments^18^ and the long-term evolution experiment (LTEE)^31–33^. Mutants that are found inside of enzymatic domains are more likely to affect protein function (Fig. 3b). Since QSPACE maps these functional and mutational datasets to protein structures, the 3D distance between mutants and enzymatic domains can identify mutants that are proximal to — but not found within — enzymatic domains.

Using the distance between annotated protein active sites and ALE mutations, we identify mutations across the *E. coli* proteome that are likely to affect protein active sites (Fig. 3c). Extending our analysis to include 1,395 subcategories of functionally important regions of the *E. coli* proteome (Dataset S4, Fig. S4), we find 2,549 instances (1,924 in ALE, 625 in the LTEE) of moderate-to-severe (Grantham Score^37^ > 100) mutations being present within 1 amino acid length of functionally important protein regions (Fig. S5 and S6, Dataset S5).

Additionally, once QSPACE establishes a structural proteome for a reference strain, the open reading frame (ORF) alleleome can be mapped in 3D to show the variable positions in all proteins across all strains of a species^34^. The interpretation of such mapping is facilitated by the fact that the ORF alleleome: i) is conserved (the majority of amino acid positions are the same), ii) is narrow (variable amino acid positions typically have only one or two alternate amino acids), and iii) the vast majority of amino acid substitutions come with low Grantham scores (i.e., protein properties are unlikely to experience significant changes).

The mapping of the conserved and narrow alleleome^34^ onto the MG1655 strain’s structural proteome effectively gives structural proteomes for a large number of *E. coli* strains. This new scale of structural proteomics (i.e., from a strain to a species) is achieved by multi-omic data integration using QSPACE’s genome-wide quaternary structures.

### The membrane module yields angstrom-level subcellular compartmentalization of the *E. coli* proteome

While mapping data types to individual protein complex structures can prove useful, understanding the location and space that these protein complexes occupy in the cell is important for building a genome-scale representation that reflects the 3D physical embodiment of a proteome. To date, genomic databases of *E. coli* (e.g., EcoCyc^38^) assign the entire gene to a subcellular compartment. UniProt^39^ sometimes offers sequence annotation of transmembrane and topological (‘bulb’) domains, however, these annotations are often inaccurate (see Fig. 4b) or missing entirely. This lack of standardization often incorrectly classifies membrane-associated proteins (proteins with no membrane-embedded region) as ‘membrane’ proteins and is not sufficient to provide the proper orientation of a protein across the cell membrane. Likewise, sequence-based prediction software (e.g., DeepTMHMM^40^) and structure-based prediction software (e.g., OPM^41^) are agnostic to membrane orientation and can also generate erroneous results.

To achieve an angstrom-level physical representation of the *E. coli* proteome, we use a structure-guided approach that combines and assesses all available annotations and predictions to correctly identify the integration and orientation of the membrane-embedded proteome. To this end, QSPACE classifies all 1.34 million codon positions of the *E. coli* proteome into one of twelve subcellular compartments.

QSPACE queries the available gene-level subcellular compartment information provided by Ecocyc^38^, UniProt^39^, Gene Ontology^42^, and genome-scale model iML1515^36^ to identify all potential membrane-embedded protein structures (Fig. 4a). For each identified structure, QSPACE determines the membrane-spanning residues for each subunit using the sequence annotations provided in UniProt, the sequence-based predictions generated by DeepTMHMM^40^, and the structure-based calculation of the membrane planes predicted by OPM^41^. For each of the three sources of residue information (when available), QSPACE calculates the normal vectors of the corresponding membrane planes (Fig. 4b). For each pair of membrane planes, the angle between the planes, and the thickness and area of the membrane-embedded region are used to determine whether the calculated membranes are viable (Fig. 4c).

QSPACE segregates each viable membrane protein into three sections: a membraneembedded region and two ‘bulbous’ regions. Each bulb is automatically assigned (Dataset S6) to either the cytoplasmic, periplasmic, or extracellular side of either the inner or outer membrane, using the annotated topological domains in UniProt or manually assigned (Dataset S7) using common 3D motifs in the protein structures (Fig. 4d). Proteins annotated to the cell membrane (Fig. 4a) that do not contain a membrane-embedded region are considered ‘membrane-associated’ and tagged to their respective membrane while those tagged to the cytoplasm or periplasm are left unchanged. The gene ontology (GO) terms of genes mapped to non-membrane proteins were used to assign proteins to the cytoplasm, periplasm, or extracellular space.

We successfully assign 86% of proteins (89% of AAs) to one of twelve subcellular compartments (Fig. 4e), resulting in an angstrom-level annotation of cellular compartmentalization of the *E. coli* proteome across both cellular membranes. The membrane integration for an additional 5% of proteins (2% of AAs) is known (Fig. 4e, #13–17), however there is insufficient information to properly orient these proteins across the membrane (see Dataset S7). This information can be combined with genome-scale computational models that compute the composition and location of the proteome, and in this manner, we can obtain a draft image of the 3D proteome of a functioning cell.

### Computing the physical space required by the *E. coli* proteome

The multi-subunit protein complexes carry out metabolic reactions, transport nutrients across the cell membrane, maintain cellular homeostasis, replicate the cellular genome, and even synthesize other proteins. Considering all these functions simultaneously calls for the use of computational models. As genome-scale models (GEMs) have increased in scope and mechanistic detail^20–24,43^, they require the biosynthesis and proper assembly of multi-subunit complexes to drive the reactions in their reconstructed metabolic networks.

While genome-scale models using protein structures (GEM-PROs) have been used for a variety of applications^44^ (e.g., contextualization of disease-associated human mutations^30^, identification of protein-fold conservation in similar metabolic reactions^29^, prediction of thermosensitivity in a metabolic network^28^, comparative structural analyses of multiple organisms^26^), the promise of a complete physical representation of a functioning cellular proteome has yet to be delivered. QSPACE moves us close to this goal by calculating the subcellular compartment of every amino acid across the proteome.

The successful annotation and 3D orientation of proteins across the subcellular compartments is a significant advancement towards building genome-scale models that can predict the physical distribution of the cellular proteome. A geometric analysis of the compartmentalized proteome (Fig. 5a) allows us to calculate the volume occupied (Fig. 5b) as well as the membrane area required (if applicable) (Fig. 5c) by each protein. Genome-scale models of metabolism and macromolecular expression (ME-models) (Fig. 5d) predict the proteome allocation required to sustain growth in optimally growing bacterial cells (Fig. 5e). In calculating the physical space required by each protein, the spatial requirements of model-predicted proteomes can also be determined (Fig. 5f–g). Thus, it is now possible to compute the composition and location of the structural proteome. A more detailed supra-protein-complexlevel 3D arrangement requires additional considerations^45–46^.

## Discussion

The 3D visualization and modeling of the structural proteome of a functioning cell has been an implicit goal of genome-scale annotations and modeling methods. QSPACE, introduced here, provides a fundamental advancement in the introduction of multi-subunit protein structures (including *de novo* annotations of protein-complex assemblies and *de novo* structural models) at the genome-scale for computational modeling and structural analyses. In conjunction with mutational databases, functional annotations, and other data types, the multi-subunit protein structures identified through QSPACE can be used to obtain a deeper understanding of whole-cell functions.

To achieve a complete physical visualization of the cellular proteome, the structure of each individual protein complex in its native oligomeric (if applicable) state is needed. To this end, QSPACE allows for multi-gene mapping to oligomeric crystallographic depositions (e.g., PDB bioassemblies), existing homo-oligomeric structural models (e.g., high-quality^47^ SWISS-PROT models^10^ with high QSPRD scores^48^), and *de novo* high quality multi-subunit structural models (from Alphafold Multimer^14^/ColabFold^35^). Unlike a purely annotative workflow, QSPACE uses a structure-guided assessment to identify previously unannotated oligomeric assemblies and generate *de novo* structural models when the existing structural data for a protein complex is incomplete. In *E. coli*, we find a 1:2 ratio between the unique genomic positions and unique proteomic positions, suggesting dimerization to be the norm (Fig. 1E). Thus, QSPACE achieves the quaternary structural representation of all multi-subunit protein complexes, a significant advancement over existing genome-scale models using structural biology software (ssbio^25^, see Fig. S1) and the gene-level monomeric structural representations available in databases (Fig. S2).

The oligomeric protein structures resolved by QSPACE offer a better-suited 3D scaffold on which to calculate protein properties, identify enzymatic domains, and analyze impactful mutations. QSPACE’s interoperability of various data types (complete list in Dataset S1), can drive biological discovery. In this study, we show how proximity-based metrics in QSPACE can be used to analyze sequence-level annotations of functional domains (e.g., protein active sites) and mutations acquired through laboratory evolution to identify mutations likely to impact protein function that may not be immediately obvious from a sequence-level analysis (Fig. 3c and S56). QSPACE can also be used to analyze the natural sequence variation of *E. coli* in three dimensions (Fig. 3a). To our knowledge, this is the first 3D representation of the natural sequence variation at the genome-scale, and it moves the description and scale of the structural proteome to the species level.

QSPACE advances whole cell modeling efforts^45,49–50^ by establishing structural annotations relevant for molecular processes. Advancements with computational genome-scale models (GEMs) over the past decade have allowed for the prediction of proteome allocation for cells at optimal growth rate^20–24,43^. Increasingly detailed, GEMs include reactions for protein assembly and translocation across subcellular compartments (e.g., membranes), however, previous GEM formulations with monomeric protein structures (GEM-PROs) have yet to reflect the full biophysical embodiment of these *in silico* processes.

Using a structures-based approach that combines and assesses all available annotations and predictions for membrane-spanning proteins, QSPACE predicts the membrane integration and orientation of proteins across both the inner and outer membrane of *E. coli*. In fact, QSPACE even calculates membrane integration for proteins that span both membranes (e.g. AcrAB-TolC efflux pump, PDB:5v5s, see Fig. 4e). As such, QSPACE provides the subcellular compartmentalization for *every amino acid* of the *E. coli* proteome. As a proof-of-concept, we combine the protein-level information in QSPACE with a genome-scale model of macromolecular expression (iJL1678b-ME^24^) to calculate the spatial requirement for the predicted proteome of *E. coli* at optimal growth rate. To our knowledge, this is the first GEM-PRO that embodies the spatial allocation of the oligomeric proteome of *E. coli*.

Taken together, QSPACE generates full quaternary proteomes for a genome (i.e., a strain) and thus provides a new level of genome annotation. It can accommodate natural sequence variations described by the recently revealed alleleome^34^ to generate species-level structural proteomes, and it enables a physical embodiment of the structural proteome against the 3D structure of the bacterial cell. As structures are resolved for large protein complexes, as the scope of genome-scale models expands to include an increasing number of niche cellular mechanisms (e.g., stress responses), and as new mutations of functional importance are annotated to publicly available databases, QSPACE will provide an interoperable pipeline for the structural proteomes for a growing list of organisms. QSPACE thus represents a notable advancement in the systems biology of structural proteomes.

## Materials and Methods

### Mapping structural data to *E. coli* genes.

#### Procurement and sequence alignment of PDB structures.

A set of 4,349 genes (Blattner Numbers) were identified in the alleleome of a collection of 2,611 wild-type *E. coli* strains. For each gene, the WT consensus amino acid sequence and details of sequence variation were determined as described previously. The UniProt^39^ search tool was used to identify the UniProt ID for 4,228 genes. With some additional manual curation, the UniProt ID was identified for a total of 4,316 genes (#000A, Dataset S8). For each gene, the UniProt entry metadata (.txt files) and the sequences (.fasta) were downloaded (#000B).

Each gene that mapped to a UniProt entry ID was also mapped to protein structures in the PDB^13^ using one of three methods (#002A). First, the PBDe-API (ssbio.databases.pdb.best_structures) was used to directly identify the best PDB chain (PDB chain with highest sequence identity) for each UniProtId. Second, the MMseqs2-based sequence search service of the RCSB PDB Search API^51^ was used to identify protein structures that shared sequence similarity to the amino acid sequences determined from UniProt (identity_cutoff = 0.20, evalue_cutoff = 0.0001). Third, wild-type consensus AA sequences were queried against the RCSB PDB (Sequence) Search API to identify additional PDB structures. We used a low cutoff of 20% sequence identity for our sequence-based queries to increase the number of structures and unique oligomeric protein conformations mapped to our gene list.

In total, we were able to identify 55,370 unique PDB entries that mapped to 3,200 genes in our gene list (Fig. S7A-B). Each PDB entry identified included at least one map between a chain on the protein structure and at least one gene in our query. We were able to download CIF files for 99.2% (54,953/55,370) of the PDB entries mapped to 96.6% (3,090/3200) of our gene set. For each of these PDB entries, all available bio-assemblies were also downloaded, resulting in 80,361 bioassembly CIF files (Fig. S7C). By parsing the CIF files in each bioassembly and its corresponding PDB CIF file, all unique oligomeric conformations of a protein structure were determined (Dataset S8, #002B) resulting in the identification of 53,310 non-monomeric conformations (Fig. S7D).

This oligomeric mapping between PDB entityIds, bioassembly entityIds, stoichiometric ratios, and structure chainIds was used to transfer the sequence alignment scores from the MMseqs2based sequence search service of the RCSB PDB Search API from the PDB structures to the bioassembly structures. We note that a chain in the bioassembly structures can be mapped to multiple genes with varying sequence identity scores. This multi-gene-to-single-chain-mapping is resolved by finding all possible combinations of genes that can recapture the chain stoichiometry present in a structure (see “calculating all possible combinations of mapped genes to protein structures”).

#### Procurement and sequence alignment homology models.

The I-TASSER^8^ model repository and metadata for *Escherichia coli* was downloaded (https://zhanggroup.org/QUARK/ecoli/ and https://zhanggroup.org/QUARK/ecoli2/). Shared UniProt IDs between our gene set and the I-TASSER metadata were used to map 4,226 ITASSER homology models to our gene set (Fig. S8B). We created an iterative algorithm that removed residues in “string-like” unfolded regions in the I-TASSER homology model files. Sequence alignment between the UniProt AA / WT AA sequences to the amino acid sequences of the protein subunits in each “trimmed” I-TASSER model was used to calculate the structure coverage (sequence identity) metric. The sequence identity of each protein subunit in the “trimmed” homology models reflects a better metric for the true structural coverage of these model predictions. We note that I-TASSER models are monomeric.

For each UniProt Id, the corresponding AlphaFold model and metadata was downloaded (https://alphafold.ebi.ac.uk/entry/{UniProtId}) from the AlphaFold Protein Structure Database^12^. Shared UniProt IDs between our gene set and the AlphaFold metadata were used to map 4,301 AlphaFold homology models to our gene set (Fig. S8C). The sequence alignment between the UniProt AA / WT AA sequences and the AlphaFold model allowed for the calculation of a structure coverage (sequence identity) metric for each chain in the AlphaFold structures. The sequence identity of each protein subunit and the b-factor average was used to determine the structure-chain quality of the mapping to our gene set. We note that AlphaFold models are monomeric.

The SWISS model repository^10^ and metadata for *Escherichia coli* was downloaded (https://swissmodel.expasy.org/repository. Shared UniProt IDs between our gene set and the SWISS metadata were used to map 5,938 SWISS homology models to our gene set (Fig. S8D). The sequence alignment between the UniProt AA / WT AA sequences and the SWISS-MODEL protein structures allowed for the calculation of a structure coverage (sequence identity) metric for each chain in the SWISS-MODELs. We note that SWISS models can be oligomeric.

#### Calculating pseudo-structures: all possible combinations of mapped genes that can recreate the protein-chain stoichiometry of the structure file.

For each protein structure or homology model, we know the sequence identity of each protein subunit-to-gene pairing (StructureId→SubUnitId→GeneId→SequenceIdentity). However, a single subunit may have been mapped to more than one gene as a result of the PDB search API’s sequence service. Therefore, we use a tree search algorithm to identify all possible combinations of “subunit-gene” pairings that can re-create the protein subunit stoichiometry.

These theoretical combinations of genes to protein structures are termed “pseudo-structures”. A “pseudo-structure” is a hypothetical combination of genes that can re-create the chain stoichiometry of the structure file. We note that only one gene is used per PDB entity ID (e.g. Entity #1 with chains A, B, and C will always result in a homotrimer). Similarly, a structure with monomeric Entities 1, 2 and 3 will always result in a hetero-trimer. The quality of each pseudostructure is determined by a weighted average of each of its subunit-gene mappings in proportion to the relative fraction of amino acids they represent in the protein. The total number of genes that can be mapped to pseudo-structures from various sources is shown in Figure S8A-E. Redundant structures are removed such that only the best quality pseudo-structures are selected for each unique structure-gene stoichiometry (Fig. S8F, Dataset S9). These best quality pseudo-structures are passed to the protein-complex to structure module.

### Procurement of enzyme-gene stoichiometries.

Gene-stoichiometries for 468 enzyme complexes in *E. coli* are annotated in EcoCyc^38^ public SmartTable “All Protein-Complexes and Their Components in *Escherichia coli* K-12 substr. MG1655”. Genome-scale model iJL1678b-ME^24^ provides gene-stoichiometries for 1,329 enzymes. The subset of 2,308 genes that are not included among the annotated enzymes from EcoCyc and iJL1678b are treated as expected monomers (Fig. S9A, Dataset S2). Of the known gene-stoichiometries of protein complexes, oligomerization is expected to be reflected in the structural data for 1,047 protein complexes (Fig. S9B).

### Accurate structural representation of multi-subunit enzymes.

#### Structure-guided re-annotation of enzyme-gene stoichiometry.

For monomeric genes (annotated and unmodeled) (Fig. S7B, grey), we identify all oligomeric instances for each gene in the pseudo-structures (hereafter referred to as ‘structures’) dataset (Module #003B). Since I-TASSER and AlphaFold models are monomeric, oligomeric instances are only found in the PDB and SWISS-MODEL structures. Oligomerization can be homomeric or heteromeric. We found structural evidence of oligomerization for 983 genes (Fig. S9C).

QSPACE uses a semi-automated QCQA pipeline to determine if the protein-gene stoichiometry should be modified to match the structural data (e.g. the protein is thought to be a monomer but the protein structures are all tetramers, Fig. 2a.ii). Oligomeric evidence derived from SWISS models failing one or more QCQA metrics (GMQE^47^ < 0.5, QMN4^47^ < −4, QSPRD^48^ < 0.5, Sequence Identity to Gene < 0.7) was removed from consideration.

The annotated gene stoichiometry for a protein-complex was automatically updated to reflect the oligomerization found in the structural data in instances where structures in the PDB and in the SWISS-MODEL repository both suggested a specific homonomeric oligomerization (*Case I*), and in instances where evidence of homonomeric oligomerization comes from exactly one SWISS model that passed QCQA (*Case II*). Instances where multiple SWISS models and PDB structures presented different homonomeric oligomeric conformations of the protein complex were decided by manual inspection of the relevant literature (*Case III*). Instances where homonomeric oligomerization (*Case IV*) or heteromeric oligomerization (*Case V*) was found in only PDB entries were also decided by manual inspection. In total, 425 monomers were reannotated as protein oligomers according to the structural data (Fig. S9D, see Dataset S2).

#### De novo structure modeling of multi-subunit enzymes using AlphaFold Multimer.

In cases where the structural data mapped to the enzyme was not sufficient to re-create the full gene-stoichiometry (Fig. 2a.iii), the amino acid sequences in the expected gene-stoichiometric ratios were sent to AlphaFold Multimer^14^ / ColabFold^35^ for *de novo* structure prediction in complexes smaller than 2000 amino acids (the computation limit for ColabFold with Google premium plus subscription) (Fig. S10A). For some large enzymes, the amino acid sequences for a subset of their protein subunits were sent to ColabFold for a more accurate structural representation (e.g. an ABC-family transporter without its periplasmic-binding domain) than what is available in the PDB or in homology model databases (e.g. individual models/structures for each subunit of the ABC-family transporter). This module was able to generate high-quality *de novo* models for 431 protein oligomers (Fig. S10B-C).

#### The matching algorithm identifies structural representations of all protein complexes.

Following the re-curation of protein complex gene stoichiometry (Fig. 2a.ii and S9) and the *de novo* generation of Alphafold Multimer structures (Fig. 2a.iii and S10), combinations of all structures (PDB, SWISS-MODEL, I-TASSER, AlphaFold in Figure S7 and AlphaFold Multimer in Figure S10) that can recreate the annotated protein-complex gene stoichiometry are found using a recursive breadth-first search tree algorithm (Fig. 2B–D) for 3,985 protein complexes.

For each protein complex, more than one structure can be used to recreate the complex’s gene stoichiometry (Fig. S11A). This is the case when a multi-subunit protein complex can be matched to a single protein structure (of the entire complex) as well as a combination of structures (of individual subunits) (Fig. 2a.i, and 2b ‘subunit’). In such cases, the single-structure representation is chosen if it is an AlphaFold Multimer model (these are already QCQA’d in Fig. S10) or if its sequence identity to the complex is greater than 70%.

For single-structure representations that fail this criteria (Fig. S11B), the choice between the single-structure representation and all multi-structure representations of the protein complex are subject to additional QCQA (Fig. S11C-E) to determine if the loss of quaternary structure is worth the increase in sequence identity provided by the multi-structure representations (representations with the maximum number of structures are made up of separate high-quality homology models for each protein subunit).

### Calculating protein properties using various software platforms.

#### Mapping of disordered regions using DisEMBL.

Amino acid sequences for each gene were sent to DisEMBL^52^. Disordered regions identified as ‘coils’ (DSSP secondary structure designations T, S, B, and I), ‘hotloops’ (coils with high degree of mobility determined by alpha-carbon B-factors), and rem465 (missing structure coordinates) were mapped to their corresponding three-dimensional coordinates.

#### Mapping protein-interfaces regions geometrically and with ScanNet.

For protein structures with multiple subunits, a geometric determination was used to identify protein-protein interfaces. The distance between the alpha-carbons of amino acid pairs belonging to different protein subunits was calculated. Distance thresholds of 3, 5, 7.5 and 10 angstroms were used to define protein-protein interfaces. For all protein structures, ScanNet^53^ was also used to predict residues involved in protein-protein binding sites.

#### Secondary structure classifier and properties.

Amino acid sequences were analyzed using SCRATCH^54^. Three-class (ss) and eight-class secondary structure elements were identified (ss8). Amino acid sequences were analyzed using DSSP^55^. Seven-class (ss) secondary structure elements were identified. IUPAC backbone torsion angles (phi/psi) were also identified. Amino acid sequences were analyzed using DisEMBL^48^. Disordered regions forming “coils” were identified (DSSP secondary structure designations T, S, B and I). UniProt secondary structure feature class was used to identify annotate helix, strand, turn and coiled regions. Sequence-level data was mapped back to the protein structures using the sequence alignment of the protein chain and gene sequence.

#### Calculation of tertiary/quaternary structure properties.

Amino acid sequences were analyzed using SCRATCH^54^. Relative solvent accessibility was calculated. Two-class solvent accessibility determination was predicted (exposed vs buried) for each residue (acc). Relative solved accessibilities were also predicted from 0–100% scale (acc20). Amino acid sequences were analyzed using DSSP^55^. The residue solvent exposed surface area was calculated (asa). The relative residue solvent exposed surface area was calculated (rsa). Protein structure files were analyzed using MSMS^56^. Residue depths (res_depth) and alpha-carbon depths (ca_depth) were calculated. Protein structures were analyzed using ssbio. Pairs of cysteines within 3 angstroms of each other (disulfide bridges) were identified. The sequence annotations in UniProt with feature class “DISULFID” was also used to identify disulfide bridges. The sequence annotations in UniProt with feature class “CROSSLNK” were used to identify residue crosslinks. Amino acid sequences were analyzed using DisEMBL^48^ where disordered regions were classified into “hotloops” (coils with high degree of mobility determined by alpha-carbon B-factors) and “rem465” (missing structure coordinates). Sequence-level data was mapped back to the protein structures using sequence alignment of the protein chain and gene sequence.

### Analysis of protein domains of enzymatic and evolutionary importance.

#### Functional protein domain annotation.

Functional domains are annotated in the “feature” (FT) class of the UniProt text files. We use the sequence alignments described previously to map the amino acids belonging to a protein domain to an amino acid in the three-dimensional structural representation. QSPACE-GS was able to identify 1,526 unique functional domains distributed across 291,674 unique genome positions (Dataset S4, Fig.S4).

#### Annotation of laboratory-acquired mutations and natural observed variants.

Also included in the feature class of the UniProt text files is information about known sequence variants (“VARIANT”) and studied mutations (“MUTAGEN”). We find 8,140 unique genome positions with annotated sequence variation in UniProt and/or mutation phenotype metadata.

ALE mutations from ALEdb^18^ were mapped to genomic positions in *E. coli* as described in Catoiu et al. 2023^34^. QSPACE-GS maps 42,785 ALE mutations to three-dimensional to their corresponding structural coordinates. ALE mutations affect 98,806 amino acids (after oligomerization) of the structural proteome.

Mutations^31^ acquired in the long term evolution experiment^32–33^ (LTEE) were mapped to genomic positions in *E. coli* as described in Catoiu et al. 2023^34^. QSPACE-GS maps 10,365 LTEE mutations to three-dimensional to their corresponding structural coordinates. LTEE mutations affect 21,299 amino acids (after oligomerization) of the structural proteome.

The WT consensus amino acid sequence and dominant amino acid occurrence was calculated as described in Catoiu et al. 2023^34^. QSPACE-GS maps the normalized occurrence of the WT dominant amino acid and the WT dominant codon to amino acids found in protein structures. Additional variant-level information is also provided.

#### Identification of mutants of interest

QSPACE can be used to identify mutants of interest in multiple ways. In this study, we show that a simple presence/absence count can be used to identify ALE mutations present in functional domains of a single protein (Fig. 3A–B). This analysis can be expanded to all proteins, to multiple protein domain types, to multiple sources of mutations, and to incorporate the annotated severity (Grantham Score^37^) of the mutation. Likewise, we show that a proximity metric can be used to identify mutants of interest. In this study, we measure the distance between each ALE mutation and its nearest active site and group the mutations by severity (Grantham score) (Fig. 3C). We expand this analysis to include different types of functional domains (Fig. S5) and to include the LTEE mutations (Fig. S6).

### Subcellular compartmentalization of the membrane proteome.

#### Identification of potential membrane structures

Blattner numbers were used to obtain gene-level subcellular information from UniProt topological annotation, EcoCyc compartment annotation, Gene Ontology (GO) terms relating to the membrane, and subcellular compartments in genome-scale model iML1515^36^.

Presence of annotated sequence features related to the membrane (TOPO_DOM, TRANSMEM, INTRAMEM) were used to identify potential membrane genes with UniProt. Genes containing membrane-related keywords (‘membrane’, ‘transport’, ‘abc’, ‘wall’ and ‘periplasm’) among their gene ontology (GO) terms were identified as potential membrane genes. These GO terms were scraped from the uniport text file for each gene. Genes containing membrane-related keywords (‘membrane’, ‘periplas’, ‘transport’, ‘secretion’, ‘extracellular’ and ‘wall’) found in the “Locations” column of the EcoCyc public SmartTable “All genes of *E. coli* K-12 substr. MG1655” were tagged to the membrane. Membrane-related keywords (‘membrane’, ‘transport’, ‘abc’, ‘wall’, ‘periplasm’, ‘envelope’ and ‘murein’) in the gene-protein-reaction (GPR) relationships from genome-scale model iML1515 were used to identify potential membrane genes.

Once the complete set of all potential membrane genes was determined, a set of protein structures selected by QSPACE whose subunits consisted of at least one membrane gene were identified as potential membrane structures (Fig. 3a). This set of structures was analyzed further to confirm membrane embeddedness.

#### Identification of membrane-embedded and membrane-crossing residues.

Membrane-embedded residues are used to define amino acids in between the two membrane leaflets. Membrane-crossing residues are used to define amino acids that are at the surface of membrane leaflets.

The membrane-embedded and membrane-crossing residues were identified using the sequence-based topological annotation provided in UniProt, a sequence-based prediction using DeepTMHMM^40^, a structure-based prediction using OPM^41^. It is important to note that membrane-embedded and membrane-crossing residues are determined on a per-gene basis for UniProt and DeepTMHMM. For proteins with multiple subunits, we map the information from these sources to each subunit to identify all membrane-embedded/crossing residues at the level of the protein-complex.

For UniProt annotations, transmembrane helices (membrane-embedded residues) are annotated as “TRANS_MEM” in the uniport.txt files for each gene. Residues at the termini of each annotated transmembrane helix (membrane-crossing residues) were assigned to their nearest topological domain. If no topological domain information was included, annotated known termini residues of helices were assigned to opposite topological domains.

Amino acid sequences for all proteins were uploaded to DeepTMHMM web server (https://dtu.biolib.com/DeepTMHMM). DeepTMHMM predicts whether a residue falls inside (membrane-embedded) or outside the membrane. DeepTMHMM is orientation-agnostic (orientation is dependent on user input). For DeepTMHMM results, the first topological “outside” domain was noted. Orientation-agnostic labels “leaflet 1” and “leaflet 2” were assigned to alternating “outside” domains (every other “outside” domain is on the same side of the membrane). Termini residues of predicted transmembrane “inside” domains (membrane-crossing residues) were assigned to their nearest (in terms of amino acid residue number) outside domain.

We uploaded the 3D coordinate files of all potential membrane structures to OPM (https://opm.phar.umich.edu/ppm_server2_cgopm) and downloaded the membrane predictions and associated PDB file with membrane atoms. OPM provides orientation-agnostic membrane-embedded residues. The termini residues of all predicted transmembrane segments (membrane-crossing residues) were assigned to their closest calculated membrane plane (‘in’ vs ‘out’). For OPM-generated PDB files, this can be done by separating membrane-crossing residues by the sign of their z-coordinates.

#### Calculation of membrane planes from membrane-crossing residues.

For each group of “*k”* membrane-crossing residues assigned to the same membrane leaflet, the 3D coordinate of each residue’s alpha-carbon was determined from the protein structure. For this set of 3D coordinates, the normal vector and error of the membrane plane was calculated for the least-squares solution. In an iterative process for groups containing more than four amino acid residues (*k>4*), each residue was sequentially removed from the group and a new membrane plane and error was calculated for the *k-1* remaining residues. The new membrane plane with the least error was chosen. The variance of the set of distances between each residue and the calculated membrane plane was determined. If the change in variance between subsequent iterations was less than 5%, the membrane plane with the least error in the last iteration was chosen as the final representation of the membrane plane. This iterative membrane calculation is especially useful in removing the errors introduced by membrane-crossing residues determined from poorly annotated transmembrane helices in UniProt.

OPM results include a PDB file with additional atoms where the membrane is predicted to be. Rather than using the membrane-crossing amino acids in the least-squares membrane calculation, we use the coordinates of a small subset of these OPM-generated atoms to calculate the normal vector of each membrane plane. These OPM-generated atoms are already coplanar, making the membrane calculation trivial. We noticed that OPM doesn’t add membrane-atoms for some PDB outputs (we believe this issue is caused by a limit on the number of atoms that can be in the PDB file output, which is encountered in the largest protein complexes). When this happens, we use the membrane-crossing residue information provided in the results.html webpage and use a least-squares calculation described above to determine the membrane plane.

An example of the membrane plane calculation for UniProt, TMHMM, and OPM is shown in Figure 4b. Additional examples can be seen in Fig. 5 and Dataset S7).

#### Determining viable membranes.

Following the calculation of membrane planes, a protein is confirmed to the membrane fraction if 1) it contains a pair of membrane planes with normal vectors oriented at an angle less than 35 degrees; 2) the average distance between the membrane planes in the protein’s membrane-embedded region is between 12 and 45 angstroms; and 3) the areas of the projections of the membrane-embedded region into both membrane planes are less than 10,000 squareangstroms (Fig 4c). When viable membrane planes are generated from more than one source of membrane-crossing residues, deference is given to planes derived from OPM, UniProt, and DeepTMHMM, respectively.

#### Orientation of the membrane-embedded proteome.

Membrane-spanning proteins contain ‘bulbous’ domains that are not membrane-embedded. The subcellular location of these bulbous domains is used to determine the membrane orientation of these proteins. QSPACE’s only source of membrane orientation information is contained in the UniProt topological domain annotation, but this information is not available for all membrane proteins.

For proteins with final membrane planes generated using OPM/TMHMM, the residues within orientation-agnostic bulbs are matched to residues in the UniProt topological domains such that there is maximum agreement between residues in OPM/TMHMM and UniProt. This orientation is performed for proteins when the UniProt topological information is available (Dataset S6, ‘orient_by = Uniprot_Passed’), even in the absence of a viable membrane plane calculation from UniProt (orient_by = ‘Uniprot_Failed’). When UniProt topological information is not available (also the case from some UniProt-generated membrane planes containing only transmembrane helix annotations), proteins were oriented manually (Dataset S6, ‘orient_by = Manually’) using recognizable protein domains (e.g. ATP-binding domains of ABC-family inner membrane transporters are located in the cytoplasm) (see Dataset S7 for complete accounting of manually oriented membrane proteins).

#### Assignment of amino acids across 12 subcellular compartments.

The *E. coli* proteome is distributed across the cytoplasm, inner membrane, periplasm, and outer membrane. Membrane-spanning proteins contain ‘bulbous’ domains that are not membrane-embedded, but are nonetheless pegged to the membrane. This allows us to consider twelve subcellular compartments: 1) extracellular; 2) extracellular bulb of an outer membrane protein; 3) embedded outer membrane; 4) periplasmic bulb of an outer membrane protein; 5) outer membrane-associated periplasmic protein; 6) periplasm; 7) inner membrane-associated periplasmic protein; 8) periplasmic bulb of an inner membrane protein; 9) embedded inner membrane; 10) cytoplasmic bulb of an inner membrane protein; 11) inner membrane-associated cytoplasmic protein; 12) cytoplasm.

The residues of oriented membrane-embedded proteins are annotated to compartments 2–4 and 8–10. For membrane proteins where orientation could not be determined (e.g. relatively symmetric putative inner membrane transporters or single pass short transmembrane helix peptides), unknown orientation compartments 13–14 and 16–17 were used. The size of these compartments is shown in Figure 4.

For the remaining 3,154 proteins, EcoCyc (location), UniProt (topological domains), GO terms, and iML1515 reaction subsystems were used to gather the protein metadata. A keyword search of the metadata classified each protein into one or more compartments: 1) cytoplasm – ‘cyto’; 2) membrane –’membrane’, ‘wall’, ‘transport’; 3) periplasm –’periplas’; 4) extracellular - ‘extracellular’. Proteins falling in only one keyword group were classified in their respective subcellular compartments (1, 6, or 12, and compartment 18 for “membrane” proteins whose structure does not contain a viable membrane embedded region). For example, proteins linked to the “membrane” and “cytoplasm” groups were labeled in group 11 (Cytoplasm, Inner Membrane-Associated). Likewise, subcellular compartments 5, 7, and 15 could be resolved. There is a subset of proteins for which no subcellular compartment or grouping could be determined (group 19). The results of QSPACE-GS’s subcellular compartmentalization module are shown in Figure 4e.

### Integration with genome-scale model iJL1678b-ME and calculation of proteomic spatial allocation requirements.

#### Geometric analysis of membrane and non-membrane proteome.

For each protein subunit, a ConvexHull algorithm was used to calculate the volume. The sum of all subunit volumes was taken to be the volume of the protein (Fig. 5b). For membrane proteins, the area formed by the projection of all subunit vertices into their nearest membrane plane was calculated (Fig. 5b–c). Since a protein’s bulbous domain can limit their packing capacity, the larger of the two projections’ areas was taken to be a protein’s membrane area requirement.

#### Quantitative composition of GEM-predicted *E. coli* proteomes.

The enzyme-gene stoichiometries of enzyme complexes in the genome-scale model of metabolism and macromolecular expression iJL1678b-ME^24^ were updated to reflect QSPACE curation. iJL1678b-ME was solved using the soplex solver to determine the flux of complex formation reactions and protein translation reactions. For each complex, the volume and membrane surface area (if available) were multiplied by the complex formation flux to obtain values for volume allocation flux and surface area allocation flux. These geometric fluxes were assigned at the gene-level in proportion with the enzyme-gene stoichiometry. We note that we distribute the geometric fluxes across gene-stoichiometry ratios such that complexes can be more easily identified when displaying the results in Proteomaps^57^ (https://www.proteomaps.net/), and that future genome-scale models should assign the geometric flux in proportion to the relative real size of the subunits in a enzyme complex. Using Proteomaps, we display the biosynthesis proteome allocation (translation flux), the volume flux, and the surface area flux of the cell membrane predicted by QSPACE/iJL1678bME at optimal growth rate (Fig. 5e–g).

### Benchmarking QSPACE against iML1515-GP.

We compare the protein structure annotations in this study against those provided by *E. coli* genome-scale metabolic model with protein structures iML1515-GP^36^. We show that QSPACE provides superior genome coverage (Fig. S1A). The amino acid content covered by iML1515 is equal to the sum of amino acid sequence lengths across 1515 genes. The QSPACE-1515 amino acid content was calculated by summing all amino acids in unique oligomeric states discovered by QSPACE structures. The amino acid content was normalized by all unique oligomeric states of all amino acids across the entire proteome of *E. coli* calculated by QSPACE (Fig. S1B).

For each gene-structure mapping provided in iML1515, the true structure-quality was determined by QSPACE (Fig. S8B-E). This was compared to the quality of the structure chosen to represent the gene in QSPACE. If a single gene is present in multiple structures (i.e. the gene is involved in multiple protein complexes), the average quality of all gene-structure matches was used. The discrepancy in structure-quality metric is most evident in genes where iML1515 selects an I-TASSER model as the protein structure (Fig. S1C) and is illustrated by detailed inspection of the structures selected for gene *dosP* in iML1515 and QSPACE-1515 (Fig. S1D).

iML1515 selects the protein structure with the highest sequence identity to a gene’s reference sequence as its structural representation, which can lead to an overestimation of structure quality. As iML1515 only maps genes to structures in the PDB and ITASSER (618 and 897 of 1515 genes, respectively), overestimation of quality in ITASSER models can be compounded. With the increased range of structure selection to include SWISS, AlphaFold, and AlphaFold Multimer models, QSPACE uses a combination of sequence identity, misfolded region penalization, AlphaFold model metrics, SWISS-model metrics, and I-TASSER model metrics to calculate quality metrics more accurately for selected protein structures.

Since QSPACE calculates the oligomeric assembly of the proteome, QSPACE-1515 can give us an improved understanding of the oligomerization states of iML1515 genes. The gene length (monomer length, in AAs) was calculated for all 1515 genes. If the gene is determined to be part of an oligomer by QSPACE-1515, the size of the oligomer was calculated. Oligomers can represent associations of the same gene (homo-oligomerization) or associations with additional genes (hetero-oligomers). In the case of hetero-oligomerization, the amino acid length(s) of the additional gene(s) in the oligomer were calculated. A 3D scatter plot of monomeric and oligomeric sizes shows that only 30% of iML1515 genes are truly monomeric (Fig. S1E). Thus, QSPACE provides a significant advancement in structural protein annotation over iML1515.

### QSPACE computation times for varying numbers of genes.

We ran QSPACE for varying gene inputs. QSPACE-24 provides an example set of 24 genes that show the functioning of various QSPACE modules (e.g. Alphafold Multimer, membrane orientation, structure-guided reannotation, etc.). QSPACE-1515 was run for the set of 1,515 iML1515 genes and used to benchmark the QSPACE structural annotation quality against the iML1515-GP structural annotations (Fig. S1). QSPACE-GS is our genome-scale QSPACE run for the entire genome of *E. coli* K-12 MG1655 (4352 genes). The completion time for QSPACE24, −1515, and -GS computations were 12 minutes, 9.2 hours, and 15.1 hours respectively. We note that these run times took advantage of previously calculated data (e.g. using previous sequence alignments rather than re-doing the alignment at every run; using previously downloaded OPM membrane calculations rather than re-uploading structures to OPM and waiting for the results; etc.), and *de novo* generation of QSPACEs (using the ‘force_rerun=True’ flag) is likely to require additional time.

## Data Availability

All data are freely available from public sources. Structures selected from the PDB were last downloaded March 5^th^, 2023. We show experimental structures from the PDB with accession numbers 2GRX, 5V5S, 7NYU, 1NEK, 6OQS, 6C53, 1PFK, 6V0C. Structures selected from the SWISS-MODEL *E. coli* repository were last downloaded December 20^th^, 2022. We show SWISS-MODELs with UniProt IDs P33232 and P39099. Structures selected from the ITASSER *E. coli* database repository were last downloaded November 3^rd^, 2022. Structures selected from the Alphafold database were last downloaded January 15^th^, 2023. We show Alphafold models with UniProt IDs P33924 and P30143. Structures were last modelled using ColabFold on April 6^th^, 2023. We show Alphafold Multimer/ColabFold models for protein complexes with EcoCyc IDs CYT-D-UBIOX-CPLX and ABC-13-CPLX. Protein complex gene stoichiometry data for *E. coli* is provided by the Public SmartTable in EcoCyc at https://ecocyc.org/group?id=Biocyc12-4862-3584200844 and by genome-scale model iJL1674b-ME at https://github.com/SBRG/ecolime. ALE mutation data is available at https://aledb.org/. LTEE mutation data is available at https://barricklab.org/shiny/LTEE-Ecoli/. Both mutation datasets were mapped to the *E. coli* genome by Catoiu *et. al*. 2023^34^. Data generated in this study is provided in the Supplementary Material and/or at https://github.com/EdwardCatoiu/QSPACE/.

## Code Availability

All source code for QSPACE is provided at https://github.com/EdwardCatoiu/QSPACE/.

QSPACE could not be possible without the following:

Python v.3.7.9 (https://www.python.org/); Ssbio v.0.9.9.8 (https://github.com/SBRG/ssbio); Biopython v.1.81 (https://github.com/biopython/biopython); ScanNet (https://github.com/jertubiana/ScanNet); Nglview v.0.11.9 (https://github.com/nglviewer/nglview); Pandas v.1.1.5 (https://github.com/pandas-dev/pandas); SciPy v.1.5.4 (https://github.com/scipy/scipy); NumPy v.1.19.5 (https://github.com/numpy/numpy); Matplotlib v.3.3.3 (https://github.com/matplotlib/matplotlib); Matplotlib_venn v.0.11.9 (https://github.com/konstantint/matplotlib-venn); PyVenn (https://github.com/tctianchi/pyvenn); Seaborn v.0.10.1 (https://github.com/mwaskom/seaborn);
